# Individualized treatment strategies for hyperuricemia informed by a semi‐mechanistic exposure‐response model of uric acid dynamics

**DOI:** 10.14814/phy2.13614

**Published:** 2018-02-27

**Authors:** Sergey Aksenov, Carl C. Peck, Ulf G. Eriksson, Donald R. Stanski

**Affiliations:** ^1^ Quantitative Clinical Pharmacology Early Clinical Development IMED Biotech Unit AstraZeneca Waltham MA; ^2^ University of California at San Francisco and NDA Partners LLC San Luis Obispo CA; ^3^ Quantitative Clinical Pharmacology Early Clinical Development IMED Biotech Unit AstraZeneca Gothenburg Sweden; ^4^ Quantitative Clinical Pharmacology Early Clinical Development IMED Biotech Unit AstraZeneca Gaithersburg MD

**Keywords:** Allopurinol, febuxostat, fractional excretion, glomerular filtration rate, gout, hyperuricemia, lesinurad, mathematical modeling, nephrolithiasis, oxypurinol, pharmacodynamics, pharmacokinetics, renal physiology, uric acid, uricosuria, uricosuric, xanthine oxidase inhibitor

## Abstract

To provide insight into pharmacological treatment of hyperuricemia we developed a semi‐mechanistic, dynamical model of uric acid (UA) disposition in human. Our model represents the hyperuricemic state in terms of production of UA (rate, *PUA*), its renal filtration (glomerular filtration rate, GFR) and proximal tubular reabsorption (fractional excretion coefficient, *FE*). Model parameters were estimated using data from 9 Phase I studies of xanthine oxidase inhibitors (XOI) allopurinol and febuxostat and a novel uricosuric, the selective UA reabsorption inhibitor lesinurad, approved for use in combination with a XOI. The model was qualified for prediction of the effect of patients' GFR and *FE* on concentration of UA in serum (sUA) and UA excretion in urine and their response to drug treatment, using data from 2 Phase I and 4 Phase III studies of lesinurad. Percent reduction in sUA from baseline by a XOI is predicted to be independent of GFR,*FE* or *PUA*. Uricosurics are more effective in underexcreters of UA or patients with normal GFR. Co‐administration of a XOI and an uricosuric agent should be considered for patients with high sUA first in the treatment algorithm of gout before uptitration of XOI. The XOI dose in combination with a uricosuric can be reduced compared to XOI alone for the same target sUA to the degree dependent on patient's GFR and *FE*. This exposure‐response model of UA can be used to rationally select the best drug treatment option to lower elevated sUA in gout patients under differing pathophysiological situations.

## Introduction

Hyperuricemia is an abnormally elevated concentration of uric acid in serum that is associated with increased risk of gout, and independently of renal and cardiovascular disease (Bardin and Richette 2014). Uric acid production is a consequence of purine degradation. While the physiological processes of uric acid disposition have been experimentally identified, their roles in the hyperuricemic state and effects of pharmacological treatment have not been previously quantitatively described.

Pathophysiological and molecular abnormalities in the processes that contribute to production and elimination of uric acid in the body are well studied and understood. Mutations have been identified that alter activity of enzymes involved in purine metabolism and result in pathological overproduction of uric acid (Seegmiller et al. 1967; Sperling et al. 1973). Overwhelming release of intracellular nucleic acids by necrotic cells can lead to increased metabolic production of uric acid, such as in tumor‐lysis syndrome associated with high concentration of uric acid in serum (Wilson and Berns 2012). Reduced glomerular filtration rate (GFR), one manifestation of impaired renal function, has been associated with hyperuricemia in physiological studies (McPhaul 1968) and epidemiological studies (Krishnan 2012). Increased serum uric acid is associated with several genetic variants including those that affect transporters involved in renal disposition of uric acid (Kolz et al. 2009). These alterations in filtration and renal disposition of uric acid can lead to lower renal excretion of uric acid, resulting in increased serum concentrations.

While there appears to be no consensus about a threshold concentration for increased risk of the various diseases linked to hyperuricemia, the general approach to management of hyperuricemia in patients with gout has been to induce a decrease in the concentration of uric acid in serum to less than 6 mg/dL and often 5 mg/dL (Khanna et al. 2012). Concentrations above this target value may exceed the solubility of uric acid (6.8 mg/dL in physiological fluids) and result in deposition of uric acid crystals in tissues and joints, which can induce acute inflammatory episodes in gout. Pharmacological interventions to decrease uric acid in serum include xanthine oxidase inhibitors that inhibit production of uric acid and uricosuric agents that increase urinary excretion of uric acid (Khanna et al. 2012). Increased excretion of uric acid by the kidney is achieved by inhibition of its reabsorption in renal proximal tubule epithelium.

In this article, we propose a semi‐mechanistic, dynamical exposure‐response model of pathophysiologic processes of uric acid production and elimination in the human body for prediction of the effect of pharmacological interventions in hyperuricemic patients. Our model provides a quantitative framework in which biomarkers reflecting pathophysiological conditions of the patient causing the altered disposition of uric acid are related to concentration of uric acid in serum and the rate of its urinary excretion–laboratory manifestations of hyperuricemia. Estimation of model parameters and qualification of the model description of uric acid disposition and predictions of drug interventions in hyperuricemic patients were performed using human experimental data from the clinical development program of lesinurad (Hoy 2016). Lesinurad is a novel selective uric acid tubular reabsorption inhibitor targeting the URAT1 transporter of uric acid and, in combination with allopurinol or febuxostat, approved in the US and Europe for patients who do not reach the target serum uric acid on xanthine oxidase inhibitors alone.

The main message of this article is that optimal application of pharmacological interventions in hyperuricemia requires consideration of key patient's pathophysiological parameters of uric acid disposition: GFR and fractional excretion coefficient of uric acid. Reliance solely upon the clinical presentation of hyperuricemia to choose doses of xanthine oxidase inhibitors and uricosuric agents does not provide sufficient information to predict decrease in serum uric acid in an individual patient. Nor can this approach provide for acceptable balance of the degree of decrease of uric acid in serum and increase of its urinary excretion, which is a goal of safe and effective treatment of hyperuricemia. The high rate of urinary excretion is one of the contributing risk factors for uric acid nephrolithiasis (Maalouf et al. 2004).

### Glossary

**Table:** 

GFR	Glomerular filtration rate (mL/min)
[*P*]_50*,*PIN_	Plasma concentration of an inhibitor of uric acid production resulting in half‐maximal fractional decrease in the production rate of uric acid (ng/mL)
*R*_max_	Maximum fractional decrease in the production rate of uric acid due to the inhibitor of uric acid production
[*P*]_50,RIN_	Plasma concentration of an inhibitor of uric acid reabsorption resulting in half‐maximal increase in the fractional excretion coefficient (ng/mL)
*F*_max_	Maximum increase in fractional excretion coefficient due to the inhibitor of uric acid reabsorption
*CL*_I_	Intestinal clearance of uric acid (L/h)
*V*_UA_	Volume of distribution of uric acid (L)
*F*_E_	Fractional excretion coefficient of uric acid in urine
*F*_E,0_	Fractional excretion coefficient of uric acid in urine without drug treatment
*S*_UA_	Amount of uric acid in serum (mg)
[*S*_UA_]	Concentration of uric acid in serum (mg/dL)
*k*_P_	Production rate of uric acid (mg/h)
*k*_P,0_	Production rate of uric acid without drug treatment (mg/h)
*X*_UA_	Excretion rate of uric acid in urine (mg/h)
*CL*_UA_	Total clearance of uric acid (L/h)
*U*_UA_	Amount of uric acid excreted in urine (mg)
[*P*]_PIN_	Plasma concentration of the inhibitor of uric acid production rate
[*P*]_RIN_	Plasma concentration of the inhibitor of uric acid reabsorption

## Mathematical Model and Methods

### A semi‐mechanistic, pathophysiological exposure‐response model of uric acid disposition

**Figure 1:**
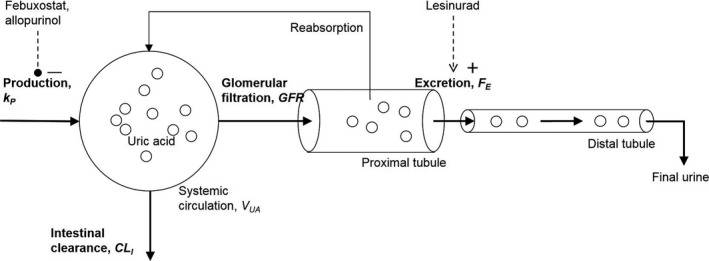
A schematic of key processes of uric acid disposition. Uric acid enters the systemic circulation by metabolic production (production rate *k*_P_). It is eliminated from the circulation by intestinal clearance and renal clearance. Renal clearance occurs by filtration in the glomeruli and as reflected by the glomerular filtration rate and partial reabsorption in the proximal tubule. The effect of reabsorption is described by the fractional excretion coefficient of uric acid (*F*_E_).

A schematic of the mechanism of key physiological processes of uric acid disposition (Hediger 2005) that are relevant to our model is shown in Figure 1. Uric acid in serum is produced by metabolic conversion from purines by xanthine oxidase during turnover of endogenous cell content and ingestion of purine containing food (Gutman 1951). Elimination of uric acid from the body occurs by excretion in the kidneys and secretion in the gastrointestinal tract (Sorensen 1965). In the kidney, uric acid is filtered through glomeruli and reabsorbed by transporters in the proximal tubule of the nephrons so that only a small fraction of the amount filtered by the glomeruli of the nephrons is excreted in urine (Bobulescu and Moe 2012). The fraction of uric acid excreted in urine is normally in the range of 7–12% in healthy adults. A major transporter for reabsorption of uric acid from the lumen of the proximal tubules is URAT1 (Enomoto et al. 2002), while the role of other transporters for UA reabsorption has also been documented (Hyndman et al. 2016).

The differential equation of the model describing the balance of rates of production and elimination of uric acid in serum, *S*_UA_ (mg), is:

(1) $$\frac{dS_{\mathrm{UA}}}{\mathrm{dt}}=k_{P}-CL_{I}\times {\left[S\right]}_{\mathrm{UA}}-\text{GFR}\times F_{E}\times {\left[S\right]}_{\mathrm{UA}}$$

The first term of Equation (1), *k*_P_ (mg/h), is the constant production rate of uric acid. The second term is the intestinal excretion rate (mg/h). It is proportional to intestinal clearance parameter *CL*_I_ (L/h) and concentration of uric acid in serum [*S*]_UA_ (mg/L). The third term is the uric acid excretion rate from the proximal tubule (mg/h). It is proportional to model parameters GFR (L/h), *F*_E_, and concentration of uric acid in serum [*S*]_UA_. The value of *F*_E_ is the fraction of excretion across all nephrons in both kidneys, which is the fraction of filtered uric acid that is not reabsorbed.

The rate of appearance of uric acid in urine *U*_UA_ (mg) equals the rate of its excretion from the proximal tubule assuming no reabsorption or secretion of uric acid downstream of the proximal tubule (all known and characterized transporters of uric acid are localized in the proximal tubule, in human (Bobulescu and Moe 2012)):

(2) $$\frac{dU_{\mathrm{UA}}}{\mathrm{dt}}=\text{GFR}\times F_{E}\times {\left[S\right]}_{\mathrm{UA}}$$

Concentration [*S*]_UA_ is defined using the ratio of *S*_UA_ and volume of distribution of uric acid, *V*_UA_ (L):

(3) $${\left[S\right]}_{\mathrm{UA}}=\frac{S_{\mathrm{UA}}}{V_{\mathrm{UA}}}$$

The error models for the difference between observed and predicted concentration of uric acid in serum [*S*]_UA_ and the amount excreted in urine *U*_UA_, both represented by *f* in the equation below, have both additive and proportional variance components:

(4) $$y=f+\sqrt{a^{2}+{\left(b\times f\right)}^{2}}\times \varepsilon$$

where *y* is the observed uric acid in serum or urine, *a* and *b* are standard deviations and *ε* is distributed independently and Normally about mean 0 with variance 1.

The concentration of uric acid in serum [*S*]_UA_ at steady state is obtained by setting the rate of change in the amount in serum in Equation (1) to zero:

(5) $${\left[S\right]}_{\mathrm{UA}}=\frac{k_{P}}{{\mathrm{CL}}_{I}}+\text{GFR}\times F_{E}$$

The rate of excretion of uric acid in urine, *X*_UA_, at steady state is then

(6) $$X_{\mathrm{UA}}=\frac{k_{P}\times \text{GFR}\times F_{E}}{CL_{I}+\text{GFR}\times F_{E}}$$

Given the concentration of uric acid in serum [*S*]_UA_ and amount excreted in urine *X*_UA_ measured without drug treatment targeting uric acid production and its fractional excretion, we can calculate the baseline values of *k*_P,0_ and *F*_E,0_ by solving the steady state Equation (5) and (6):

(7) $$k_{P,0}={\left[S\right]}_{\mathrm{UA}}\times CL_{I}+X_{\mathrm{UA}}$$

and

(8) $$F_{E,0}=\frac{X_{\mathrm{UA}}}{{\left[S\right]}_{\mathrm{UA}}\times \text{GFR}}$$

### Models of the effect of drug interventions on parameters of uric acid disposition

Relative decrease in the production rate of uric acid with respect to an untreated value by a production rate inhibitor was modeled as a saturable function of drug concentration in plasma [*P*]_PIN_ achieving a finite maximum at large concentration of the drug:

(9) $$k_{P}=k_{P,0}\times \left(1-\frac{R_{\max}\times {\left[P\right]}_{\mathrm{PIN}}}{{\left[P\right]}_{\mathrm{PIN}}+{\left[P\right]}_{50,\mathrm{PIN}}}\right)$$

*R*_max_ is the nondimensional maximum relative decrease in the production rate due to the drug and [*P*]_50,PIN_ is plasma concentration of drug resulting in half‐maximal relative decrease in the rate. The rate at maximal effect of the drug is *k*_P,0_ × (1–*R*_max_), while a concentration of drug in plasma equal to [*P*]_50,PIN_ it is *k*_P,0_ × (1–*R*_max_/2).

The increase in the fractional excretion coefficient of uric acid *F*_E_ by a uric acid reabsorption inhibitor was modeled as a saturable function of drug concentration in plasma [*P*]_RIN_ (concentration units depend on the drug):

(10) $$F_{E}=\frac{F_{E,0}+F_{\max}\times {\left[P\right]}_{\mathrm{RIN}}}{{\left[P\right]}_{\mathrm{RIN}}+{\left[P\right]}_{50,\mathrm{RIN}}}$$

Here *F*_max_ is the nondimensional maximum increase in the *F*_E_ due to the drug and [*P*]_50,RIN_ is plasma concentration of the drug resulting in half‐maximal increase in *F*_E_. Fractional excretion coefficient of uric acid at maximal effect of the drug is *F*_E,0_ + *F*_max_¸ while at the concentration of drug in plasma equal to [*P*]_50,RIN_ it is *F*_E,0_ + *F*_max_/2

The pharmacokinetic models that were used in this article to simulate drug concentration [*P*]_PIN_ and [*P*]_RIN_ for estimation of model parameters are given in Appendix 1.

### Data used for estimation of model parameters and model qualification

**Figure 2:**
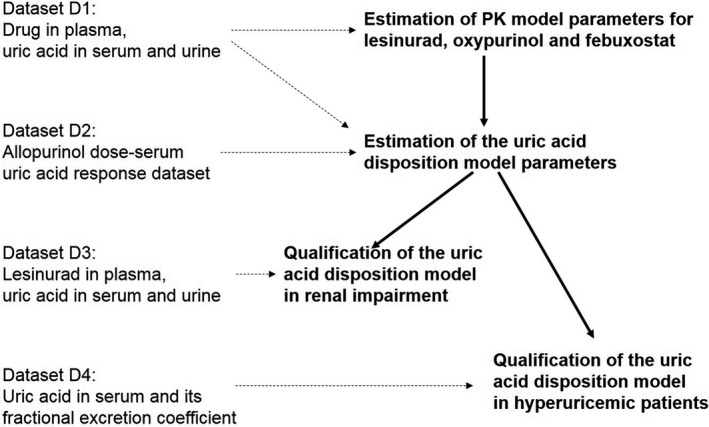
A schematic of estimation of model parameters and model qualification. In the first step parameters of the PK models were estimated. Simulated PK model parameters were used as input into estimation of uric acid model parameters. The model was qualified using independent Phase I data under renal impairment and Phase III data in hyperuricemic patients. Datasets that inform each step of modeling are shown on the left. PK, pharmacokinetic.

The datasets used for estimation of model parameters and model qualification are summarized in Figure 2. See Appendix 3 for a description of clinical studies and the data.

The dataset “D1” for estimation of parameters of the uric acid disposition model (eq. 1 and 2), models of the effect of drug concentration on uric acid model parameters (eq. 9 and 10) and pharmacokinetic models (eq. A1) comprised data from 278 subjects from nine Phase I studies of lesinurad (Fleischmann et al. 2014; Shen et al. 2015; and unpublished trial data, Ardea Biosciences). These subjects received single and multiple daily doses of lesinurad between 50 and 1600 mg and lesinurad in combination with allopurinol (300 mg) or febuxostat (40 or 80 mg). These data comprised serial measurements of concentration of lesinurad, oxypurinol and febuxostat, concentration of uric acid in serum and the amount of uric acid excreted in urine. We used the concentration of oxypurinol for modeling because it is the active metabolite of allopurinol that contributes the majority to allopurinol's activity (Day et al. 2007).

The dataset “D2” was used to estimate parameters of the effect of oxypurinol on production of uric acid because it employed a wide range of allopurinol doses. The data comprised individual predose concentration of uric acid in serum in 8 healthy subjects who received daily doses of allopurinol between 50 and 900 mg (Graham et al. 1996).

The dataset “D3” for qualification of the model of uric acid disposition in subjects with reduced GFR comprised individual serial measurements of lesinurad concentration, concentration of uric acid in serum and the amount of uric acid excreted in urine in 39 renal impaired subjects from two Phase I studies 104 and 120 (unpublished trial data, Ardea Biosciences). Subjects in Study 104 received a single dose 200 mg of lesinurad. Subjects in Study 120 received a single dose 400 mg of lesinurad. The range of GFR in the two studies was 22–164 mL/min.

The dataset “D4” for qualification of the model in patients with hyperuricemia comprised individual values of relative change from baseline of serum uric acid and change from baseline of the fractional excretion coefficient of uric acid in 647 subjects in four Phase III studies of lesinurad in patients with hyperuricemia: Study 301 (Saag et al. 2017), Study 302 (Bardin et al. 2017), Study 304 (Dalbeth et al. 2015) and Study 303 (unpublished trial data, Ardea Biosciences, ClinicalTrials.gov identifier NCT01508702). Subjects in the active arms of Study 301 and 302 were given lesinurad 200 mg or 400 mg plus a medically appropriate dose of allopurinol between 200 and 900 mg once a day depending on the local label. The vast majority of subjects received 300 mg once a day, subjects in Study 304–lesinurad 200 mg or 400 mg plus febuxostat 80 mg, once a day, and Study 303–lesinurad 400 mg once a day. Individual subject data were means of all available monthly on‐treatment values of serum uric acid and the fractional excretion coefficient.

Concentrations of lesinurad, febuxostat, and oxypurinol in blood plasma were measured up to 24 h after single and repeated dosing in the Phase I studies. Concentration of uric acid in serum was measured up to 24 h before and after drug administration in the Phase I studies, and once during monthly clinical visits in the Phase III studies. Concentration of uric acid in urine was measured in 6‐h urine collection intervals over 24 h before and after drug administration in the Phase I studies. Interval collection of uric acid in urine was not performed in the Phase III studies. The amount of uric acid excreted in urine during each interval was calculated by multiplying the concentration of uric acid in the urine sample by the volume of urine over the interval.

The concentrations of lesinurad and febuxostat in plasma samples were assayed using high‐performance liquid chromatography with tandem mass spectrometric detection (LC‐MS/MS) by Ardea Biosciences (San Diego, California). The concentrations of oxypurinol in plasma samples were quantified using LC‐MS/MS by Anapharm, Inc. (Québec, Canada). The concentrations of uric acid in serum and in urine samples were quantified using enzymatic assays by different vendors depending on the study: Anapharm, Inc. , ICON Central Laboratories (Farmingdale, NY), or Covance Central Laboratory (Indianapolis, Indiana).

### Estimation of model parameters

Parameters of the models were estimated in the sequence shown in Figure 2. The individual subject data in dataset “D1” were divided into 18 treatment groups corresponding to different doses of lesinurad alone or in combination with allopurinol or febuxostat (Appendix 3) to estimate the mean model parameters. Individual dynamic responses and the resulting time profiles of uric acid concentration in serum were sufficiently similar to justify modeling the mean dynamics of uric acid in serum and urine.

First, parameters of the pharmacokinetic models (eq. A1) for lesinurad, febuxostat and oxypurinol were estimated for each group. Next, parameters of the uric acid disposition model Equations (1, 2, 9 and 10) were estimated. During estimation, drug concentrations for each treatment group in the dataset were simulated with the corresponding pharmacokinetic models using the estimated parameter values to provide input to Equations (9 and 10) to calculate *k*_P_ and *F*_E_ in the presence of drugs. Parameters GFR and *F*_E,0_ were set to mean of measured individual values in each group. GFR was approximated with creatinine clearance calculated using the Cockcroft‐Gault formula and actual body weight without adjustment or normalization. Baseline *F*_E,0_ was calculated with Equation (8) using measured predose concentration of uric acid in serum, GFR and excretion rate in urine. The value of *k*_P,0_ for each treatment group was recalculated during each iteration of the parameter estimation routine using Equation (7), given the current value of the estimated parameter *CL*_I_.

The value of *R*_max_ for febuxostat was fixed at 1 (Bhattaram and Gobburu 2017) and *R*_max_ for oxypurinol–at a value estimated in Appendix 2 using dataset “D2”. We did not estimate *R*_max_ for febuxostat and oxypurinol using our data because in our data the range of doses (300 mg allopurinol and 40 or 80 mg febuxostat) was not sufficiently wide to reliably estimate this parameter. The value of *F*_max_ for lesinurad was fixed at a value estimated using a subset of the data corresponding to a wide range of lesinurad doses given alone. Parameters [*P*]_50,PIN_ for febuxostat and oxypurinol and [*P*]_50,RIN_ for lesinurad were estimated separately for gout subjects with hyperuricemia in Study 110 and 111 and subjects in other studies, as was done in the pharmacokinetic‐pharmacodynamic model of febuxostat (Bhattaram and Gobburu 2017).

### Adequacy of the model

Adequacy of the model was adjudged using the residual plots of differences between observed and model‐predicted concentrations of uric acid in serum and excretion rates in urine. A model was considered to adequately describe the data if trends of residuals versus time and predicted values did not deviate from random patterns, and parameters were estimated with relative standard error of less than about 30%.

### Model qualification under reduced glomerular filtration rate

Qualification of the model description of the role of GFR in uric acid disposition and model predictions of therapeutic interventions in hyperuricemic patients with reduced GFR was performed by comparing model predictions of concentration of uric acid in serum and the amount of uric acid excreted in urine with observations in subjects with reduced GFR in Study 104 and 120 (dataset “D3”). Predictions were made using final parameter estimates (value of lesinurad [*P*]_50,RIN_ for hyperuricemic patients).

Model predictions of the dependence on GFR of predose, steady‐state concentration of uric acid in serum and its excretion rate in urine to compare with the observed value were made using Equations (5) and (6). Parameters *k*_P_ and *F*_E_ in these equations were obtained by interpolation with nonlinear regression of individual subject values *k*_P,0_ and *F*_E,0_ versus GFR_._ The individual values were calculated for each subject with Equations (7) and (8) using pretreatment GFR*,* concentration of uric acid in serum and its excretion rate in urine. The nonlinear regression equations were: in Study 120 *F*_E,0_ = 0.06 + 0.15 × exp(−0.06 × GFR) and *k*_P,0_ = 47–55 × exp(−0.06 × GFR); in Study 104 *F*_E,0_ = 0.07 (does not change with GFR) and *k*_P,0_ = 37–2 × exp(0.015 × GFR).

The interpolated values were used in Equations (1, 2, and 10) to calculate the dependence on GFR of predicted changes of concentrations of uric acid in serum and excretion rates in urine relative to the pretreatment values induced by single doses of lesinurad. Time profiles of plasma concentration of lesinurad in individual subjects were interpolated with a spline function (i.e., a piecewise polynomial) over observed lesinurad concentration values and used as input into Equation (10) to calculate the change in fractional excretion coefficient of uric acid in the presence of lesinurad. Model Equations (1 and 2) for each subject were simulated over 24 h and mean concentration of uric acid in serum and cumulative excretion of uric acid in urine over 24 h after dose were calculated to then calculate the predicted relative change with respect to the pretreatment values. The observed change in fractional excretion coefficient of uric acid was calculated as difference of the coefficient after the single dose of lesinurad, *F*_E,post_ and the predose value *F*_E,0_. The value of *F*_E,post_ was calculated using Equation (8) with using pretreatment GFR*,* 24‐h mean of concentration of uric acid in serum postdose and excretion rate in urine obtained by dividing total uric acid excreted in urine by 24 h.

### Model qualification for patients with hyperuricemia

Qualification of the model predictions of therapeutic interventions in patients with hyperuricemia was performed by comparing individual predicted and observed relative change in serum uric acid from baseline in a subset of subjects in the Phase III Study 301, 302, 303, and 304 (dataset “D4”). The selected subjects' serum uric acid decreased from baseline and the fractional excretion coefficient–increased, leaving out those who did not comply with treatment (i.e., increased serum uric acid from baseline) and those who had erroneous measurement of the fractional excretion coefficient (i.e., decreased fractional excretion). For calculation of the observed relative change, the baseline value of serum uric acid for each subject was the observed value. The value of serum uric acid on treatment was the mean of the observations from all available visits for each subject.

For calculation of the predicted relative change, the baseline value of serum uric acid [*S*]_UA_^BASE^ was set to the observed value. The predicted relative change in serum uric acid from baseline was calculated as:

(11) $$\frac{\Delta {\left[S\right]}_{\mathrm{UA}}}{{\left[S\right]}_{\mathrm{UA}}}{|}_{\mathrm{Pred}}=\frac{{\left[S\right]}_{\mathrm{UA}}^{\mathrm{ON}}-{\left[S\right]}_{\mathrm{UA}}^{\mathrm{BASE}}}{{\left[S\right]}_{\mathrm{UA}}^{\mathrm{BASE}}}$$

The value of serum uric acid on treatment [*S*]_UA_^ON^ for each subject was calculated with Equation (5). In this equation, *F*_E_ was equal to the measured value of fractional excretion coefficient on treatment with lesinurad. The value of *k*_P_ was calculated with Equation (7) using baseline GFR and *F*_E_.

For subjects receiving allopurinol at baseline (Study 301 and 302), we recalculated the value of the production rate of uric acid on treatment with lesinurad to reflect the effect of co‐administered lesinurad on clearance of oxypurinol. Lesinurad at 200 and 400 mg coadministered with 300 mg allopurinol decreased daily average concentration of oxypurinol by 32% (Perez‐Ruiz et al. 2016). The corrected relative change in serum uric acid from baseline can be calculated by considering the change due to a lower concentration of oxypurinol following Equation (9):

(12) $$\begin{array}{cc} & \frac{{\left[S\right]}_{\mathrm{UA}}^{\mathrm{CORR}}-{\left[S\right]}_{\mathrm{UA}}^{\mathrm{ON}}}{{\left[S\right]}_{\mathrm{UA}}^{\mathrm{ON}}}=\\ & \left(1-\frac{R_{\max}\times 0.68\times {\left[P\right]}_{\mathrm{PIN}}}{0.68\times {\left[P\right]}_{\mathrm{PIN}}+{\left[P\right]}_{50,\mathrm{PIN}}}\right)/\left(1-\frac{R_{\max}\times {\left[P\right]}_{\mathrm{PIN}}}{{\left[P\right]}_{\mathrm{PIN}}+{\left[P\right]}_{50,\mathrm{PIN}}}\right) \end{array}$$

where [*P*]_PIN_ is average daily concentration of oxypurinol without lesinurad. The corrected change when allopurinol is coadministered with lesinurad is

(13) $$\begin{array}{c} \begin{array}{cc} & \frac{\Delta {\left[S\right]}_{\mathrm{UA}}}{{\left[S\right]}_{\mathrm{UA}}}{|}_{\mathrm{Corr}}\\ & =\frac{{\left[S\right]}_{\mathrm{UA}}^{\mathrm{CORR}}-{\left[S\right]}_{\mathrm{UA}}^{\mathrm{CORR}}}{{\left[S\right]}_{\mathrm{UA}}^{\mathrm{BASE}}}=\frac{{\left[S\right]}_{\mathrm{UA}}^{\mathrm{CORR}}}{{\left[S\right]}_{\mathrm{UA}}^{\mathrm{ON}}}\times \frac{{\left[S\right]}_{\mathrm{ON}}^{\mathrm{UA}}}{{\left[S\right]}_{\mathrm{BASE}}^{\mathrm{UA}}}-1\\ & =\frac{{\left[S\right]}_{\mathrm{UA}}^{\mathrm{CORR}}}{{\left[S\right]}_{\mathrm{ON}}^{\mathrm{BASE}}}\times \left(\frac{\Delta {\left[S\right]}_{\mathrm{UA}}}{{\left[S\right]}_{\mathrm{UA}}}{|}_{\mathrm{Pred}}+1\right)-1\\ & =\left(1-\frac{R_{\max}\times 0.68\times {\left[P\right]}_{\mathrm{PIN}}}{0.68\times {\left[P\right]}_{\mathrm{PIN}}+{\left[P\right]}_{50,\mathrm{PIN}}}\right)/\left(1-\frac{R_{\max}\times {\left[P\right]}_{\mathrm{PIN}}}{{\left[P\right]}_{\mathrm{PIN}}+{\left[P\right]}_{50,\mathrm{PIN}}}\right)\\ & \times \left(\frac{\Delta {\left[S\right]}_{\mathrm{UA}}}{{\left[S\right]}_{\mathrm{UA}}}{|}_{\mathrm{Pred}}+1\right)-1 \end{array} \end{array}$$

In this equation mean concentration of oxypurinol in plasma on 300 mg allopurinol daily [P]_PIN_ is about 10,000 ng/mL (Study 110, unpublished trial data, Ardea Biosciences). This value corresponds to 35% decrease in serum uric acid, similar to the observed value 34% (Schumacher et al. 2008).

### Prediction of the effect of drug interventions on serum uric acid reduction

Predictions of the response of serum uric acid and its excretion in urine following drug interventions to inhibit the production rate of uric acid and increase its fractional excretion coefficient were performed using simulation of Equations (5) and (6) on a grid of percent decrease in *k*_P_ and the increase in *F*_E_.

The baseline value of serum uric acid for calculations of relative change was an input parameter. The baseline urine excretion rate was then calculated using Equation (6). For a given baseline concentration of serum uric acid and a combination of GFR and baseline *F*_E_, the production rate *k*_P_ was calculated using Equation (5).

### Software for estimation of model parameters and model simulation

Parameters of the uric acid disposition model parameters and pharmacokinetic models were estimated using NonMem 7.3.0 (Icon Development Solutions, Ellicott City, MD). Dataset preparation, model simulation and preparation of all graphics were performed using R 3.2.4 (R‐project, www.r-project.org). Simulation of differential equations was performed with the R package deSolve version 1.14 with default settings which provides an interface to the ordinary differential equation solver lsoda (Hindmarsh 1983). Parameters of the oxypurinol model in Appendix 2 were estimated using the Levenberg‐Marquardt algorithm (Moré 1978) implemented in the R package minpack.lm version 1.2 with default settings. NonMem and R were run on a x86 64‐bit CentOS version 6.8 platform.

## Results

### Estimation of model parameters

Parameter values of the uric acid disposition model and models of the effect of lesinurad, febuxostat and oxypurinol on the production rate and fractional excretion coefficient of uric acid estimated using clinical data in subjects with normal GFR are shown in Table 1. Parameters were estimated with adequate precision with relative standard error less than 30%.

**Table 1:** Model parameters estimated using clinical data

Parameter	Value	Standard error (%)
*CL*_I_ (L/h)	0.27	16
*V*_UA_ (L)	19	4
*R*_max_ (oxypurinol)	0.84	NA
*F*_max_ (lesinurad)	0.56	NA
*R*_max_ (febuxostat)	1	NA
[*P*]_50,PIN_ (oxypurinol, ng/mL)	14,000	11
[*P*]_50,RIN_ (lesinurad, not hyperuricemia, ng/mL)	11,000	4.7
[*P*]_50,RIN_ (lesinurad, hyperuricemia, ng/mL)	23,000	7.1
[*P*]_50,PIN_ (febuxostat, not hyperuricemia, ng/mL)	87	9.6
[*P*]_50,PIN_ (febuxostat, hyperuricemia, ng/mL	120	10
Standard deviation, additive measurement error, serum UA	0.45	16
Standard deviation, proportional measurement error, serum UA	0.15	8.8
Standard deviation, additive measurement error, urine UA	50	8
Standard deviation, proportional measurement error, urine UA	0.29	4.2

NA, standard error for *F*_max_ and *R*_max_ was not calculated because these parameters were fixed during estimation.

**Figure 3:**
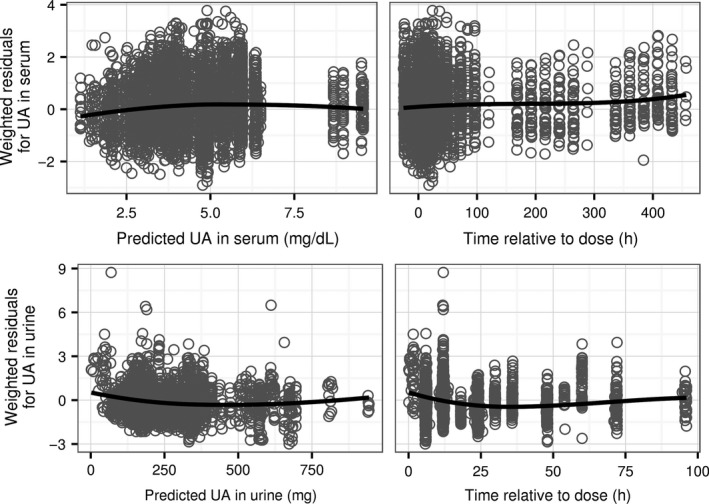
Deviations of model predictions from observations of UA in serum and urine in the dataset used for estimation of model parameters expressed as weighted residuals, that is, differences between predictions and observations divided by the estimated standard deviation of the measurement error (4455 serum samples and 3058 urine samples). Top row: residuals for concentration of UA in serum. Bottom row: residuals for the amount of UA in urine. Symbols: values of residuals. Lines: trend lines calculated as a smoothed conditional mean of the residuals. UA, uric acid.

Deviations of model predictions from the observed concentrations of serum uric acid and its excretion rate in urine are randomly distributed regardless of the time elapsed after drug dose or the magnitude of the prediction (Fig. 3). The random patterns indicate that the model adequately describes the observed data in all studies using different doses of drugs and as well as for single and multiple doses of drugs.

**Figure 4:**
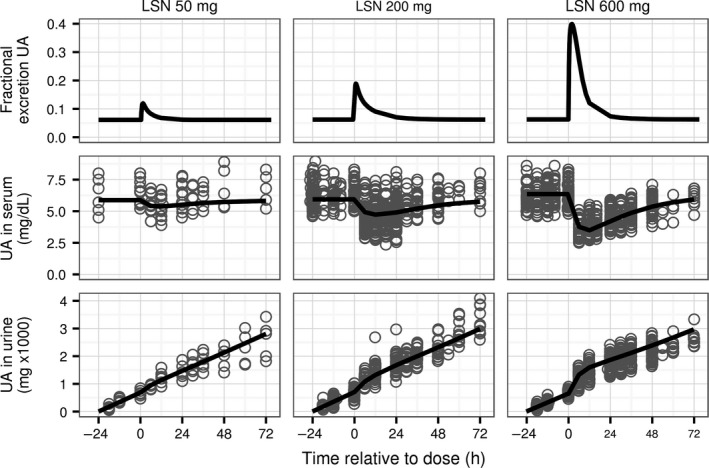
Time profiles of predicted and observed concentration of UA in serum and cumulative amount in urine for three single doses of lesinurad in studies 101, 102, 103, 109, and 125 (72, 540, and 519 serum samples and 63, 280, and 426 urine samples for 50, 200, and 600 mg, respectively). Top row: predicted fractional excretion coefficient of UA in urine. Middle row: concentration of UA in serum. Bottom row: cumulative amount of UA in urine. Horizontal axis shows time after drug dose. Values with negative time are observations and predictions before the dosing. Symbols: individual observations. Lines: model predictions using estimated parameter values. UA, uric acid. LSN, lesinurad.

The model adequately reflects dynamic responses of uric acid in serum and urine to single and multiple doses of lesinurad and allopurinol or febuxostat. Agreement between observations and predictions of the model using estimated parameter values after administration of three different single doses of lesinurad, chosen to span the dose range in the clinical data, is shown in Figure 4. After a dose of lesinurad the predicted fractional excretion coefficient of uric acid increases over time to its maximum value and then decreases back to its original value following the time profile of lesinurad concentration in plasma. The indirect effects of lesinurad are transient decreases of the concentration of uric acid in serum, and increases of slope of the time curve for cumulative uric acid in urine. The effects of lesinurad on concentration of uric acid in serum and its excretion rate in urine increased with the dose of lesinurad.

**Figure 5:**
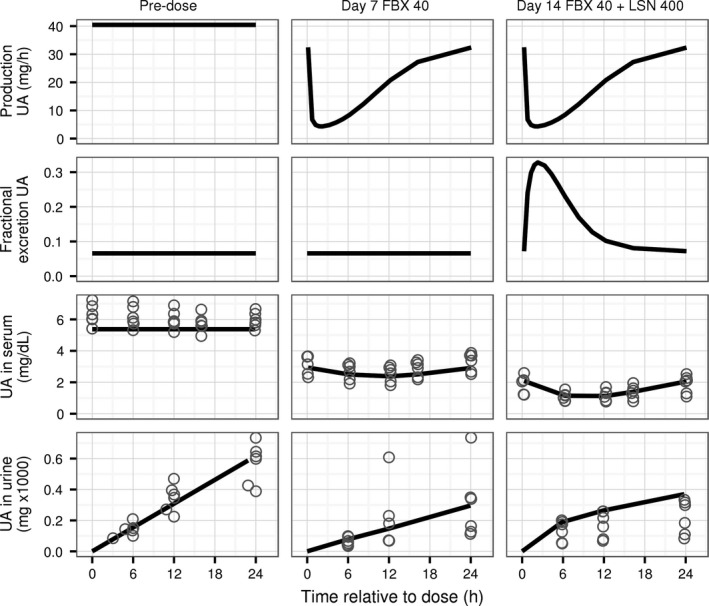
Time profiles of predicted and observed concentration of UA in serum and cumulative amount excreted in urine for daily doses of febuxostat and lesinurad in Study 105 (90 serum samples and 54 urine samples). Left column: predose. Middle column: profiles after 7 days of febuxostat 40 mg once a day. Right column: profiles after 7 days of febuxostat 40 mg and lesinurad 400 mg once a day. Panels across columns show data for the same group of patients switching treatments every 7 days. Top row: predicted production rate of UA. Second row: predicted fractional excretion coefficient of UA in urine. Third row: concentration of UA in serum. Fourth row: cumulative amount of UA in urine (amount was reset to 0 at the start of sampling on day 7 and 14). Horizontal axis shows time after drug dose. Symbols: individual observations. Lines: model predictions. UA, uric acid. LSN, lesinurad. FBX, febuxostat.

Visual agreement between observations and predictions of the model using estimated parameter values after administration of multiple doses of febuxostat and lesinurad in combination in Study 105 is shown in Figure 5. In the treatment period of the study shown in the figure, subjects received daily febuxostat for 7 days, followed by a combination of febuxostat and lesinurad for another 7 days. After a dose of febuxostat the predicted production rate of uric acid decreases over time to a minimum value and then increases up to its original value following time profile of febuxostat in plasma. The rate of uric acid excretion in urine is lower when uric acid production rate is inhibited with febuxostat compared to the rate in the absence of febuxostat (see the lower slope of the cumulative uric acid in urine curve on febuxostat). The excretion rate increases transiently when fractional excretion coefficient of uric acid is increased with lesinurad simultaneously with inhibition of its production rate with febuxostat.

### Independent model qualification under reduced glomerular filtration rate

The altered steady state serum uric acid and response to drug treatment in a hyperuricemic patient with renal impairment is a common clinical scenario. The model correctly reflects the steady state behavior and dynamic responses of uric acid in serum and urine to single doses of lesinurad observed in subjects with reduced GFR. The data from these subjects in Study 104 and 120 were not used for estimation of model parameters (Table 1), enabling qualification of the model to describe the role of GFR in disposition of uric acid and model predictions of the effect of uricosurics on serum and urine uric acid, using an independent dataset.

**Figure 6:**
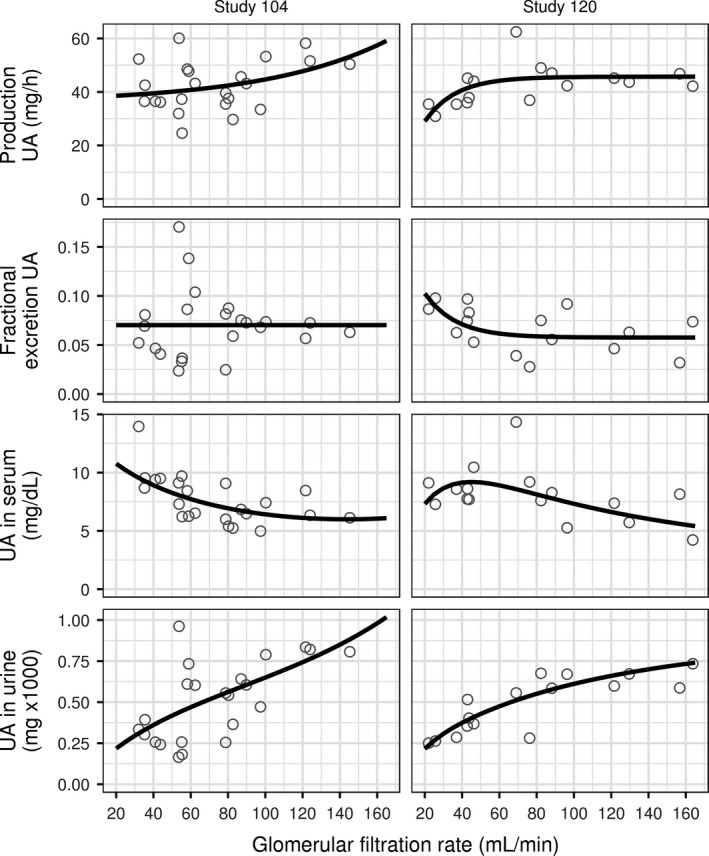
Dependence on glomerular filtration rate of predose steady state concentration of UA in serum (third row from the top) and amount of UA in urine excreted over 24 h (bottom row). Baseline production rate of UA is shown in the top row, baseline fractional excretion coefficient of UA – in the second row from the top. Left column: Study 104 (23 values). Right column: Study 120 (16 values). Symbols: values for individual subjects. Solid lines in the top two rows: interpolation of individual values using nonlinear regression. Solid lines in the bottom two rows: model predictions using interpolated mean production rate and baseline fractional excretion coefficient. UA, uric acid.

On average, steady state concentrations of uric acid in serum decrease with increasing GFR (third row from the top, Fig. 6), while the amount of uric acid excreted in urine in 24 h increases (bottom row, Fig. 6). The relationships between GFR and uric acid in serum and urine at steady state agree with observations after accounting for variation in the calculated individual production rate and baseline fractional excretion coefficient of uric acid (see Methods) among subjects in the studies (top row and the second row from the top, Fig. 6).

**Figure 7:**
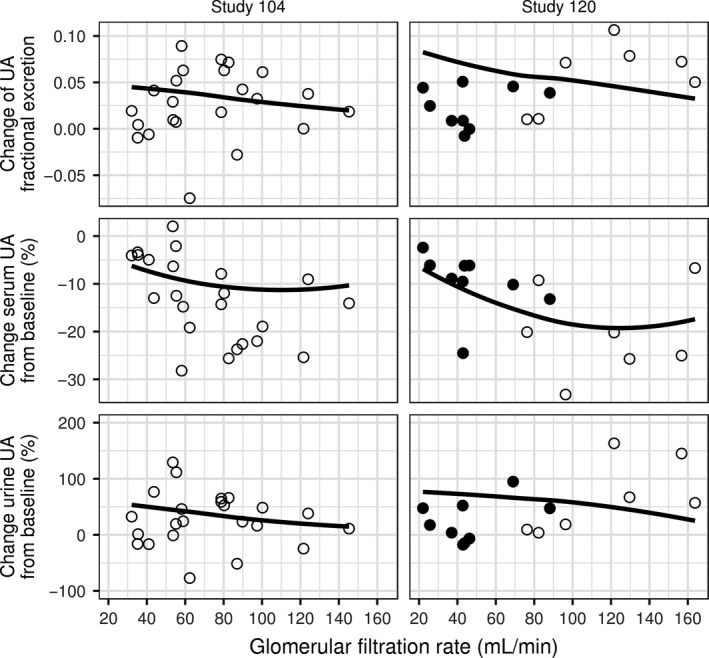
Dependence on glomerular filtration rate of response to lesinurad: 24‐h mean of change in fractional excretion coefficient of UA (top row), relative change from baseline of 24‐h mean concentration of UA in serum (second row), and relative change in the amount of UA excreted in 24 h (second row). Left column: Study 104 (23 values, single dose 200 mg). Right column: Study 120 (16 values, single dose 400 mg). Symbols: observed values for individual subjects. Filled symbols: subjects who took aspirin or insulin. Lines: smoothed conditional mean of model predictions for individual subjects, using interpolated values of baseline fractional excretion coefficient and production rate of UA that depend on GFR. UA, uric acid.

The model predictions of the relative change from baseline of the 24‐h mean serum uric acid in response to a single dose of lesinurad agree with observations in that the change is on average smaller at lower GFR values than for subjects with normal GFR in each study (solid lines, second row, Fig. 7). Relative change in the 24‐h cumulative amount of uric acid excreted in urine is on average larger at lower glomerular filtration rates (solid lines, third row, Fig. 7). The top row of the figure shows mean change in the fractional excretion coefficient of uric acid from its predose value because of the effect of lesinurad.

The model prediction tends to overpredict the magnitude of relative change in serum and urine uric acid in subjects with lower GFR in Study 120 (filled symbols). These subjects, unlike the rest of the subjects in studies 104 and 120, took either low‐dose aspirin or insulin which are known to reduce excretion of uric acid (Yu and Gutman 1959; Quiñones Galvan et al. 1995; Anzai and Endou 2012; Toyoki et al. 2017) and possibly interfere with the action of lesinurad. Aspirin at low doses was shown to decrease the effect of uricosuric probenecid on serum and urine uric acid (Yu and Gutman 1959), and to facilitate uric acid reabsorption by URAT1 (Anzai and Endou 2012) thus potentially counteracting the inhibitory effect of lesinurad on reabsorption. This may explain the model overprediction of change in fractional excretion coefficient of uric acid and the serum and urine responses to lesinurad.

### Independent model qualification in patients with hyperuricemia

**Figure 8:**
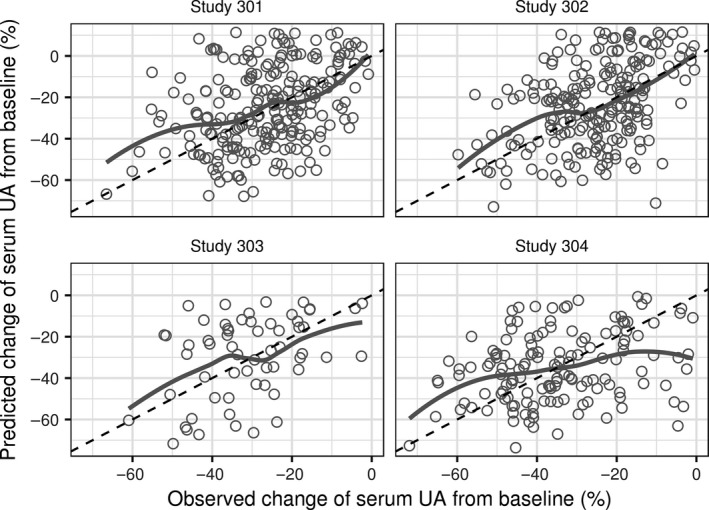
Predicted versus observed relative change in serum UA. Panels: Study 301 (230 values), 302 (216 values), 303 (60 values), and 304 (141 values). Symbols: values for individual subjects. Solid gray lines – smoothed conditional mean of predictions versus observed values. Dashed diagonal lines – reference lines where predicted and observed values are equal. UA, uric acid.

Prediction of relative change in serum uric acid from baseline in individual patients with hyperuricemia agrees with the observed values (Fig. 8). While variability in the measured serum uric acid responses is high, the predicted versus observed values cluster along the line of identity. Thus, the model is qualified to predict the dependence of serum uric acid response on baseline GFR and fractional excretion coefficient in individual patients with hyperuricemia.

### Application of the model to predict the efficacy of drug interventions to reduce hyperuricemia in patients

**Figure 9:**
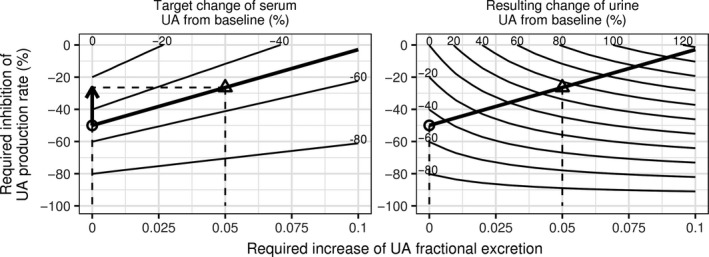
A nomogram of percent inhibition of the UA production rate and increase in UA fractional excretion coefficient in combination required to produce a target percent change in serum UA (left panel) while resulting in percent change in the amount of UA excreted in urine (right panel). Combinations of drug interventions leading to specified percent change in serum and urine UA are shown as contour lines. Increasing the degree of fractional excretion increase results in a lower required inhibition of the production rate, for the same target serum UA change. Thick lines correspond to the target decrease in serum UA by 50% from baseline. Circles indicate a scenario of inhibition of the production rate alone (50%). Triangles indicate a scenario of a combination of increase in fractional excretion by 0.05 and inhibition of the production rate by 26%. Vertical dashed lines indicate increase of fractional excretion for the two scenarios. Horizontal lines indicate inhibition of the production rate for the two scenarios. The arrow indicates the reduced inhibition of the production rate in the combination scenario. The GFR is 60 mL/min. The baseline fractional excretion coefficient is 0.03. Baseline serum UA was 12 mg/dL. UA, uric acid.

The model of uric acid disposition allows quantification of the synergy of the combined effects of inhibition of the production rate of uric acid and increase in its fractional excretion coefficient. The model can thus be used to predict effectiveness of drug interventions in patients with a range of GFR and fractional excretion coefficient. Prediction of the reduction in serum uric acid from baseline for a range of magnitudes of drug interventions is shown in the left panel of Figure 9 for a patient with baseline serum uric acid 12 mg/dL and GFR 60 mL/min and baseline fractional excretion coefficient 0.03. For a given target serum uric acid reduction from baseline (e.g., 50%), inhibition of the production rate alone needs to be 50%. Adding an intervention to increase fractional excretion coefficient allows a smaller inhibition of the production rate (follow the 50% line in the figure towards the upper right corner and read off the percent rate inhibition on the vertical axis). For example, adding an increase in fractional excretion coefficient by 0.05 requires only 26% inhibition of the production rate. The corresponding increase in urine excretion of uric acid compared to baseline is shown in the right panel of Figure 9.

**Figure 10:**
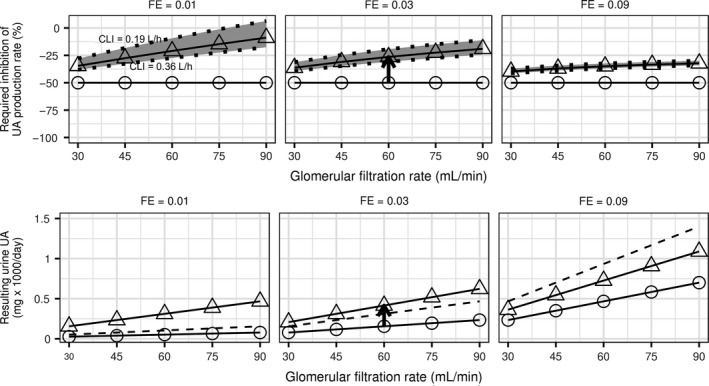
Dependence of the magnitude of inhibition of the production rate of UA in combination with increase in FE of UA required to produce 50% reduction in serum UA on baseline GFR and FE coefficient (top row). The bottom row shows the resulting amount of UA excreted in urine. Panels show simulations for baseline FE 0.01, 0.03, and 0.09. Circles correspond to inhibition of the production rate alone, triangles correspond to concomitant increase in FE by 0.05. The pair of circle and triangle connected by the arrow corresponds to the map in Figure 9. Dashed lines in the bottom row panels indicate UA excreted in urine at baseline before interventions with the production rate and FE. The grey band and dotted lines correspond to simulations for intestinal clearance *CL*_I_ in the range 0.19–0.36 L/h. The solid lines correspond to simulations with the mean *CL*_I_ = 0.27 L/h. Baseline serum UA was 12 mg/dL. UA, uric acid; FE, fractional excretion.

The magnitude of synergy varies according to the baseline GFR and fractional excretion coefficient. The top row in Figure 10 shows that the advantage of combining inhibition of the production rate with an increase in fractional excretion coefficient (vertical distance between circles and triangles in the figure) increases with GFR, and is also more prominent at lower baseline fractional excretion coefficients. The advantage of the combined treatment remains in a sensitivity analysis for a range 0.19–0.36 L/h of the intestinal clearance parameter CL_I_, corresponding to ± about 30% of the estimated value 0.27 L/h. This range is the 95% confidence interval of the mean estimate and represents a plausible range of the mean *CL*_I_ given the data used for estimating model parameters. The predicted required inhibition of the production rate of uric acid varies most in the range of *CL*_I_ at lower values of baseline fractional excretion coefficient where intestinal clearance plays greater role in the combined renal and intestinal clearance of uric acid.

Treatment decisions about the combined treatment should also consider excretion of uric acid in urine, compared to both the untreated baseline excretion and excretion on the inhibitor of the production rate alone. While the amount of uric acid excreted in urine in 24 h is always greater with than without an intervention to increase fractional excretion, it decreases with baseline GFR (bottom row in Fig. 10). The amount excreted in urine on the combination treatment is higher than the baseline excretion at lower baseline fractional excretion coefficient values, while in absolute terms the excreted amounts are low. At higher fractional excretion coefficient values the amount excreted on treatment becomes lower than the baseline excreted amount, while in absolute terms the amount excreted in urine increases. The amount of uric acid excreted in urine does not depend on the value of intestinal clearance of uric acid which is reflected in its lack of sensitivity to *CL*_I_.

## Discussion

### The model is qualified to predict therapeutic outcomes in patients with hyperuricemia

The semi‐mechanistic exposure‐response model of uric acid disposition and therapeutic intervention was developed using data rich enough to represent a wide range of perturbations of the processes of uric acid disposition and fully inform the model. Fidelity of the model to the underlying physiological processes of uric acid disposition is attested by agreement with the training data. In particular, the parallel elimination of uric acid via intestinal and renal routes incorporated in the model accounts for the dependence of baseline serum and urine uric acid and their response to lesinurad on the GFR, as confirmed with an independent dataset from two studies in renal impaired subjects.

The ability of the model to predict serum and urine uric acid responses in individual patients with a range of GFR and baseline fractional excretion coefficient, which are key patient parameters determining uric acid disposition, was confirmed with an independent dataset from three Phase III studies in hyperuricemic patients. This supports using the model to develop individualized treatment strategies.

Providing an indirect qualification of our model to describe the role of physiological parameters in disposition of uric acid, parameter values of the model are consistent with available data. The largest fractional excretion of uric acid in urine is 0.63, according to our model (*F*_max_ + typical untreated coefficient 0.07 in a non‐hyperuricemic person). It is consistent with mean 0.72 (*N* = 4) measured in Iraqi Jews with homozygous R406C or G444R loss of function mutations in URAT1 (Dinour et al. 2011), and 0.7 (*N* = 31) in Japanese hypouricemic subjects with homozygous G774A loss of function mutation in URAT1 (Ichida et al. 2008). Volume of distribution of uric acid, 19 L, is consistent with the range 14–27 L estimated with a one‐compartment model in subjects on dialysis (Ziółko et al. 2000). Mean proportion of renal clearance of the total (renal and intestinal) clearance in healthy volunteers was 0.65 (Bianchi et al. 1979), compared to 0.56–0.67 in the subjects on single doses of lesinurad (calculated using values in Table 5 in Appendix 3).

### Implications of the model for selecting therapeutic strategies

Application of the model of uric acid disposition under various therapeutic circumstances clarifies and provides mechanistic rationale for the current empirical approaches to pharmacological treatment of hyperuricemia associated with gout. We focus on gout because hyperuricemia is the main risk factor for gout. The guidelines developed by the American College of Rheumatology (Khanna et al. 2012) prior to the approval of lesinurad recommended titration of xanthine oxidase inhibitors to their maximum appropriate dose before considering a uricosuric agent alone or in combination with a xanthine oxidase inhibitor. The panel did not make recommendations for specific patient groups with respect to GFR or any other parameters, aside from suggesting a contraindication for uricosurics in patients with elevated urine uric acid excretion because of risk of urolithiasis. While consideration of patient's etiology of hyperuricemia for the choice of appropriate therapy was proposed (Emmerson 1991), it was based on categorizing patients into four discrete patterns of measurements of uric acid in serum and urine relative to prespecified normal ranges: endogenous overproduction of uric acid, exogenous overproduction because of excess purine consumption, underexcretion with normal renal function and reduced renal function. It was proposed that xanthine oxidase drugs are appropriate for patients with overproduction of uric acid, while uricosurics–for patients with underexcretion of uric acid (Emmerson 1991).

The key insight into our model that represents patients as a range of GFR and fractional excretion coefficient, rather than the above discrete subgroups or a single population, is to consider a combination therapy upfront instead of waiting to see the results of maximum uptitration of a xanthine oxidase inhibitor. At a given dose of a xanthine oxidase inhibitor the final serum concentration of uric acid depends only on its pretreatment concentration and is independent of the values of pathophysiologic parameters GFR, fractional excretion coefficient of uric acid and its production rate. For example, at 300 mg/day of allopurinol, the highest dose commonly used in clinical practice, the production rate of uric acid and thus the decrease in serum uric acid from baseline is approximately 35%. This means that on average subjects with untreated concentration of uric acid in serum >9 mg/dL and normal GFR will not reach the target of 6 mg/dL, yet these patients have the highest prevalence of gout comorbidities (Zhu et al. 2012) and are most in need of effective therapeutic intervention. Adding a uricosuric to a xanthine oxidase inhibitor in such patients will allow a substantial number of patients to reach the therapeutic target. Nomograms such as in Figure 9 can be easily constructed for each patient (given their GFR and fractional excretion coefficient) and used to determine the appropriate dose of a xanthine oxidase inhibitor, for a given uricosuric dose. For example, a nomogram for lesinurad may incorporate the average increase in fractional excretion coefficient by about 0.05 for the approved dose of lesinurad 200 mg/day.

The model shows the importance of considering individual physiology of patients to determine the xanthine oxidase inhibitor dose across patient “parametypes”–regions in the space of patient's parameters GFR and fractional excretion coefficient. Parametypes are a model representation of the physiological systems of hyperuricemia, an intermediate level of abstraction between genotype and phenotype. We have shown that this way of representing patients with hyperuricemia is useful not only to understand how parametypes determine clinical presentations of serum uric acid and urine excretion rates, but importantly, predict uric acid responses to drug treatment. Patients who have higher GFR or lower baseline fractional excretion coefficient of uric acid attain greater decrease in serum uric acid at the same dose of the reabsorption inhibitor and xanthine oxidase inhibitor than patients with reduced GFR or higher fractional excretion coefficient.

A consequence of inhibiting reabsorption of uric acid is an increase in the amount of uric acid excreted in urine over the pretreatment value, even in the presence of a xanthine oxidase inhibitor which offsets the increase in urinary excretion of uric acid. The nomograms in Figures 9 and 10 can be used to determine the appropriate allowed uric acid excretion rate in urine, considering other factors of nephrolithiasis such as baseline uric acid excretion, urine pH and flow. Larger excretion rates of uric acid in urine are expected for patients with higher fractional excretion coefficient of uric acid, who are also expected to attain greater decrease in uric acid in serum.

### Points to consider

The impacts of GFR and fractional excretion coefficient on uric acid disposition by the kidney are general and not limited to data generated using particular drugs. Nor are the insights about treatment of hyperuricemia limited to gout patients whose clinical data were employed in building and testing the model.

The model and nomograms generated with the model can be easily used in clinical practice to individualize pharmacological treatment by considering patient's GFR and fractional excretion coefficient of uric acid in making treatment decisions. Serum uric acid concentration pretreatment is routinely collected to guide pharmacological treatment. Fractional excretion coefficient of uric acid can be calculated from patient's GFR, serum uric acid and 24‐h urine excretion of uric acid using Equation (8). While the model and nomograms can be used to individualize treatment using calculated relative changes in the production rate and fractional excretion coefficient, using estimates of daily average plasma concentration of a drug, greater precision can be attained by extending the model calculations to include pharmacokinetic models of these drugs. More generally the model can be extended to any new inhibitor of uric acid reabsorption or production only by estimating parameters of the models in Equations (9) and (10) describing drug effect on the uric acid dynamics.

Our model reveals aspects of uric acid disposition and therapeutic intervention that have not been considered in their entirety by previous models. Fanelli and Weiner (1979), following Wesson (1954), developed a model of uric acid reabsorption along the length of the proximal tubule of primates that is a more detailed description of only renal disposition of uric acid. Other authors (Dua et al. 2014; Soto et al. 2015; Yoon et al. 2015) presented complementary approaches that are more detailed and focused on describing synthesis of uric acid. Our model represents a clinically useful abstraction of transport processes described by more detailed kidney models. Our model focuses on the whole‐body consequences of transport of uric acid by the human kidney, whereas models (Layton 2011; Layton et al. 2016) focus on luminal and epithelial concentrations in kidney tubules as a result of transport of a number of solutes except uric acid, in the rat kidney. In our model, we describe transport of uric acid in the human kidney with an aggregate parameter: fractional excretion coefficient. This coefficient represents the net effect of the uric acid transporters on both luminal and apical side of the proximal tubule, in human.

## Conclusions

Different xanthine oxidase inhibitors are each equally effective across the physiological ranges of renal glomerular function, fractional excretion coefficient of uric acid or production rate of uric acid. In contrast, uricosuric agents are more effective in patients who are underexcreters of uric acid or have normal GFR, according to our model. This suggests that the treatment algorithm in the gout treatment guidelines may be optimized to improve individual patient outcomes. Patients with especially high serum uric acid concentrations who are initiating therapy would likely not reach therapeutic targets even with appropriate uptitration as recommended by the gout treatment guidelines. This group of patients may benefit from more frequent monitoring of serum uric acid and earlier addition of a uricosuric agent, perhaps even at initiation of therapy. The potential benefits of such a therapeutic strategy would have to be weighed with the risks associated with the uricosuric agent. This semi‐mechanistic exposure‐response model of uric acid can be used to rationally select the best drug treatment option to lower elevated serum uric acid in gout patients under differing patho‐physiological situations.

## Conflict of Interest

SA, UE and DS are employees of AstraZeneca. CP was a consultant to AstraZeneca at the time the work was performed.

## Figures and Tables

**Figure 11:**
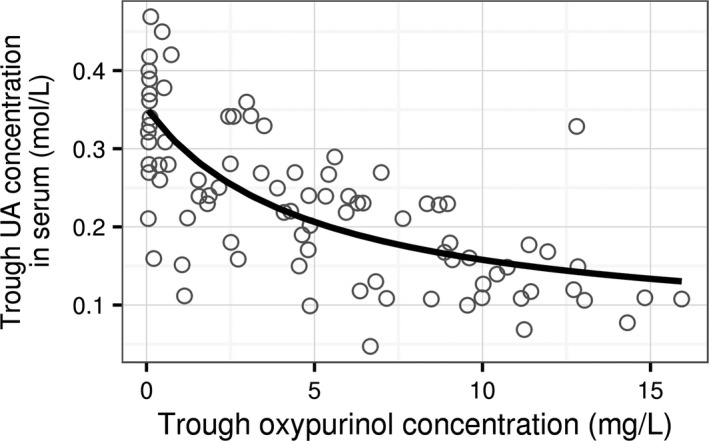
Simulation of mean trough serum uric acid concentration after 7 daily doses of allopurinol using estimated model parameters (line) and observed values (symbols).

## Appendix 1 Pharmacokinetic models

Plasma concentration of febuxostat, oxypurinol, and lesinurad were described using two‐compartment pharmacokinetic models. Equations for the rates of change in the amount of drug in the dose depot site *P*_1_, central distribution compartment *P*_2_ and peripheral distribution compartment *P*_3_ are as follows:

(A1) $$\begin{array}{cc} & \frac{dP_{1}}{\mathrm{dt}}=-k_{A}\times P_{1}\\ & \frac{dP_{2}}{\mathrm{dt}}=k_{A}\times P_{1}+\frac{Q}{V_{3}}\times P_{3}-\frac{Q}{V_{2}}\times P_{2}-\frac{\mathrm{CL}}{V_{2}}\times P_{2}\\ & \frac{dP_{3}}{\mathrm{dt}}=\frac{Q}{V_{2}}\times P_{2}-\frac{Q}{V_{3}}\times P_{3} \end{array}$$

Initial conditions for the equations if the dose was given at time 0 are:

(A2) $$P_{1}\left(0\right)=0,\;P_{2}\left(0\right)=0,\;P_{3}\left(0\right)=0$$

Drug dose *D* was applied as a bolus in the dose depot compartment:

(A3) $$P_{1}\left(0\leq t<t_{\mathrm{lag}}\right)=0,\;P_{1}\left(t_{\mathrm{lag}}\right)=D$$

Drug concentration in plasma was calculated as follows:

(A4) $$\left[P\right]=\frac{P_{2}}{V_{2}}$$

to the central compartment *k*_A_ (1/h), time of drug dose input into the system relative to dose administration time *t*_lag_ (h), clearance between the central and peripheral drug distribution compartments *Q* (L/h), drug clearance for elimination from the central compartment *CL* (L/h), apparent volume of distribution of the central compartment *V*_2_ (L) and volume of distribution of the peripheral compartment *V*_3_ (L).

The measurement error model for the difference between the observed and modeled drug concentration in plasma [*P*] had both additive and proportional variance components:

(A5) $$y=\left[P\right]+\sqrt{a^{2}+{\left(b\times \left[P\right]\right)}^{2}}\times \varepsilon$$

where *y* is the observed drug concentration in plasma, *a* and *b* are standard deviations and *ε* is distributed independently and Normally about mean 0 with variance 1.

## Appendix 2 Estimation of the effect of oxypurinol on production of uric acid

Parameters [*P*]_50,PIN_ and *R*_max_ of the model describing inhibitory effect of oxypurinol on the production rate of uric acid (Eq. 9) were estimated using paired predose values of plasma oxypurinol and serum uric acid concentrations measured in individual subjects (dataset “D2”).

Model prediction of predose concentration of uric acid serum versus predose plasma oxypurinol to calculate the sum of squared deviations of predictions from observed values was made as follows. Plasma concentration of oxypurinol versus time was simulated for a dose of allopurinol given daily using the pharmacokinetic model of oxypurinol with mean parameter values of the model (Anzai and Endou 2012; Wright et al. 2013). Simulated oxypurinol concentrations were used to calculate the production rate of uric acid over time using Equation (6). The oxypurinol pharmacokinetic model and the uric acid model Equation (1) were simulated for 7 daily doses of allopurinol and values of oxypurinol concentration and concentration of uric acid in serum 24 h after the seventh dose were recorded. This simulation was repeated for allopurinol doses 0–840 mg daily with step size 40 mg that represent the range of oxypurinol concentrations in the data. The resulting set of 22 data points were connected by linear interpolation to form the model prediction for parameter optimization.

Parameters of the uric acid disposition model and values of covariates for the oxypurinol pharmacokinetic model were fixed at values shown in Table 2 that represent healthy subjects in the study and result in pretreatment value of serum uric acid concentration similar to the mean value in the data.

**Table 2:** Parameter values that were fixed when estimating parameters of the model describing inhibitory effect of oxypurinol on the production rate of uric acid

Parameter	Value
*k*_*P*,0_ (mg/h)	50.5
*F*_E,0_	0.07
*V*_UA_ (L)	20
*CL*_I_ (L/h)	0.3
GFR (mL/min)	130
Body weight (kg)	75
Body‐mass index (kg/m^2^)	22
Sex	Male

Values of estimated parameters are shown in Table 3. Precision of parameter estimates and agreement with data are adequate with relative standard error less than 50%. Predicted serum uric acid versus oxypurinol concentration using estimated values of model parameters agree with the data (Fig. 11).

**Table 3:** Estimates of parameter values of the model describing inhibitory effect of oxypurinol on the production rate of uric acid

Parameter	Estimate	Standard error (%)
*R*_max_	0.84	15
[*P*]_50,PIN_ (mg/L)	6.4	36

Approximate 95% confidence intervals were calculated using estimated standard errors.

## Appendix 3 Data for estimation of model parameters and model qualification

The dataset “D1” for estimation of model parameters comprised data from nine Phase I studies of lesinurad. Study 101 was a sequential, single dose 5–600 mg of lesinurad study in healthy volunteers. Study 102 was a sequential, multiple dose 100–600 mg lesinurad study in healthy volunteers. Study 103 was a cross‐over, single dose 50 or 200 mg lesinurad study in healthy volunteers. Study 109 was a cross‐over, single dose 200–600 mg lesinurad study in healthy volunteers. Study 117 was a multi‐period, single dose 400–1600 mg lesinurad study in healthy volunteers. Study 125 was a single and multiple dose 50–600 mg study in healthy Japanese subjects. Study 110 was a multiple dose 400 or 600 mg/day lesinurad alone or in combination with 300 mg/day allopurinol in gout patients with hyperuricemia. Study 105 was a multiple dose 200 or 400 mg/day lesinurad alone or in combination with 40 mg/day of febuxostat in healthy volunteers. Study 111 was a multiple dose 400 or 600 mg/day lesinurad alone or in combination with 40 or 80 mg/day of febuxostat in gout patients with hyperuricemia.

Time profiles for concentration of uric acid in serum, concentration of lesinurad in plasma and cumulative amount of uric acid in urine from each subject from the nine studies were arranged in 18 treatment groups corresponding to different doses of lesinurad alone or in combination with allopurinol or febuxostat (Table 4).

**Table 4:** Treatment groups in the data set used for estimation of the model

Group	Studies	Description	Number of time profiles	Number of serum samples	Number of urine samples
LSN 50 mg	125	Single dose lesinurad 50 mg	11	72	78
LSN 100 mg	101, 102, 125	Single dose lesinurad 100 mg	26	212	202
LSN 200 mg	101, 102, 103, 109, 125	Single dose lesinurad 200 mg	90	540	506
LSN 400 mg	101, 102, 109, 125	Single dose lesinurad 400 mg	84	852	681
LSN 600 mg	101, 102, 109, 125	Single dose lesinurad 600 mg	48	519	426
LSN 800 mg	117	Single dose lesinurad 800 mg	9	141	81
LSN 1200 mg	117	Single dose lesinurad 1200 mg	10	160	90
LSN 1600 mg	117	Single dose lesinurad 1600 mg	10	160	89
Study 105‐PPB	105	Placebo qd days 1–7, febuxostat 40 mg qd days 7–21	6	210	72
Study 105‐PPA	105	Febuxostat 40 mg qd days 1–14, placebo qd days 14–21	6	210	72
Study 105‐P1A	105	Febuxostat 40 mg days 1–7, febuxostat 40 mg + lesinurad 200 mg qd days 7–14, lesinurad 200 mg qd days 14–21	6	210	72
Study 105‐P2A	105	Febuxostat 40 mg days 1–7, febuxostat 40 mg + lesinurad 400 mg qd days 7–14, lesinurad 400 mg qd days 14–21	6	210	72
Study 105‐P1B	105	Lesinurad 200 mg qd days 1–7, febuxostat 40 mg + lesinurad 200 mg qd days 7–14, Febuxostat 40 mg days 14–21	6	210	72
Study 105‐P2B	105	Lesinurad 400 mg qd days 1–7, febuxostat 40 mg + lesinurad 400 mg qd days 7–14, Febuxostat 40 mg days 14–21	6	191	66
Study 110‐P1	110	Allopurinol 300 mg qd days 1–7, allopurinol 300 mg + lesinurad 400 mg qd days 7–14, lesinurad 400 mg qd days 14–21	10	160	119
Study 110‐P2	110	Allopurinol 300 mg qd days 1–7, allopurinol 300 mg + lesinurad 600 mg qd days 7–14, lesinurad 600 mg qd days 14–21	10	153	114
Study 111‐P1	111	Febuxostat 40 mg qd days 1–7, febuxostat 40 mg + lesinurad 400 mg qd days 7–14, febuxostat 40 mg + lesinurad 600 mg qd days	12	138	138
Study 111‐P2	111	Febuxostat 80 mg qd days 1–7, febuxostat 80 mg + lesinurad 400 mg qd days 7–14, febuxostat 80 mg + lesinurad 600 mg qd days	9	107	108
Total	101, 102, 103, 109, 117, 125, 110, 105, 111		365	4455	3058

qd, once a day.

Table 5 shows mean values of predose concentration of uric acid in serum, excretion rate of uric acid in urine, subject body weight and the mean GFR and fractional excretion coefficient in each treatment group.

**Table 5:** Values of uric acid parameters, GFR and body weight prior to start of drug treatment in each treatment group

Group	Estimated GFR (mL/min)	Fractional excretion of UA, *F*_E_	Concentration of UA in serum (mg/dL)	Excretion rate of UA in urine (mg/day)	Body weight (kg)
LSN 50 mg^a^	133	0.061	6.5	648	68
LSN 100 mg^a^	128	0.063	5.9	696	74
LSN 200 mg^a^	132	0.062	6.3	672	82
LSN 400 mg^a^	116	0.073	6	696	79
LSN 600 mg^a^	113	0.063	6.3	648	81
LSN 800 mg^a^	128	0.081	4.1	600	71
LSN 1200 mg^a^	126	0.078	4.1	552	73
LSN 1600 mg^a^	108	0.108	4.3	720	67
Study 105‐PPB^a^	110	0.064	5.7	528	75
Study 105‐PPA^a^	113	0.07	5.8	600	76
Study 105‐P1A^a^	115	0.079	5.3	672	82
Study 105‐P2A^a^	121	0.066	6	576	80
Study 105‐P1B^a^	110	0.082	5.6	648	73
Study 105‐P2B^a^	137	0.054	6.7	648	84
Study 110‐P1^b^	151	0.033	9.5	696	106
Study 110‐P2^b^	129	0.037	8.9	624	101
Study 111‐P1^b^	114	0.039	8.7	552	103
Study 111‐P2^b^	133	0.043	10	744	106

GFR, glomerular filtration rate; UA, uric acid.

a Subjects without hyperuricemia.

b Subjects with hyperuricemia.

The dataset “D2” for estimation of allopurinol effect on the production rate of uric acid comprised 86 pairs of predose values of plasma oxypurinol and serum uric acid concentrations measured in 8 individual subjects receiving after 7 days of daily doses of allopurinol between 50 and 900 mg. The data were digitized from Figure 3 in (Graham et al. 1996).

The dataset “D3” for model qualification under reduced GFR comprised serial measurements of lesinurad concentration, concentration of uric acid in serum and the amount of uric acid excreted in urine in subjects with normal and impaired renal function in Study 104 (23 subjects receiving a single dose 200 mg of lesinurad) and Study 120 (16 subjects receiving a single dose 400 mg of lesinurad).

The dataset “D4” for qualification of the model in patients with hyperuricemia comprised monthly measurements of serum uric acid and in urine (to calculate fractional excretion coefficient) in four studies 301, 302, 303 and 304. The studies randomized subjects with hyperuricemia on a stable dose of allopurinol (Study 301 and 302) and febuxostat (Study 304) to once daily dose of placebo, lesinurad 200 mg and 400 mg as add‐on to allopurinol (Study 301 and 302) or febuxostat 80 mg (Study 304). Subjects were randomized to placebo or lesinurad 400 mg once daily in Study 303. There were 803 subjects in the fours studies who were randomized to active arms and had at least one value of serum uric acid and fractional excretion coefficient on treatment. Excluding subjects with increase in serum uric acid on treatment and those with a decrease in the fractional excretion coefficient from baseline, there were 647 subjects in the analysis dataset. Number of subjects and mean values of serum uric acid, GFR and fractional excretion coefficient are summarized in Table 6.

**Table 6:** Summary of Phase 3 studies used for model qualification

Study	Treatment	Number of subjects	Baseline GFR (mL/min)	Baseline FE	Change FE	CIlo change FE	CIup change FE	Baseline sUA (mg/dL)	Change sUA (%)
301	L200 + A300	113	88	0.033	0.043	0.037	0.048	7.1	−23
301	L400 + A300	117	85	0.037	0.067	0.058	0.076	6.8	−30
302	L200 + A300	108	91	0.034	0.044	0.037	0.051	6.7	−23
302	L400 + A300	108	88	0.034	0.058	0.049	0.068	6.8	−28
304	L200 + F80	64	80	0.029	0.046	0.038	0.054	5.2	−27
304	L400 + F80	77	86	0.031	0.067	0.057	0.078	5.2	−42
303	L400	60	83	0.039	0.056	0.042	0.07	9.5	−31

Treatment: L200 (lesinurad 200 mg once a day); L400 (lesinurad 400 mg once a day); A300 (allopurinol 300 mg once a day; the vast majority of patients received this dose); F80 (febuxostat 80 mg once a day).

GFR, glomerular filtration rate. FE, fractional excretion coefficient of uric acid in urine. CIlo and CIup, lower and upper endpoints of the 95% confidence interval; sUA, serum uric acid.
